# Dr. Morris J. Asch

**Published:** 1902-11

**Authors:** 


Dr. Morris J. Asch, of New York, died at Irvington-on-Hudson, October 5, 1902, aged 69 years. He graduated at Jefferson Medical College in 1855, and served in the civil war in the medical corps of the U. S. army. He resigned his army commission about 1870 and located in New York, where he became celebrated as a laryngologist. He served as a surgeon to the New York Eye and Ear Infirmary, to the Manhattan Eye and Ear Hospital and for some years was professor of diseases of the nose and throat at the New York Polyclinic. He was a member of the local medical societies and of the American Laryngological Association.

Dr. Asch was a man of charming manners, of sympathetic nature and inspired confidence among his patients who, with a large circle of friends, will ever cherish his memory.

